# Peptidic inhibitors of insulin-degrading enzyme with potential for dermatological applications discovered *via* phage display

**DOI:** 10.1371/journal.pone.0193101

**Published:** 2018-02-15

**Authors:** Caitlin N. Suire, Sarah Nainar, Michael Fazio, Adam G. Kreutzer, Tara Paymozd-Yazdi, Caitlyn L. Topper, Caroline R. Thompson, Malcolm A. Leissring

**Affiliations:** 1 Institute for Memory Impairments and Neurological Disorders (UCI MIND), University of California Irvine, Irvine, California, United States of America; 2 Department of Neurobiology and Behavior, University of California Irvine, Irvine, California, United States of America; 3 Department of Pharmaceutical Sciences, University of California Irvine, Irvine, California, United States of America; 4 Department of Chemistry, University of California Irvine, Irvine, California, United States of America; CHA University, REPUBLIC OF KOREA

## Abstract

Insulin-degrading enzyme (IDE) is an atypical zinc-metalloendopeptidase that hydrolyzes insulin and other intermediate-sized peptide hormones, many of which are implicated in skin health and wound healing. Pharmacological inhibitors of IDE administered internally have been shown to slow the breakdown of insulin and thereby potentiate insulin action. Given the importance of insulin and other IDE substrates for a variety of dermatological processes, pharmacological inhibitors of IDE suitable for topical applications would be expected to hold significant therapeutic and cosmetic potential. Existing IDE inhibitors, however, are prohibitively expensive, difficult to synthesize and of undetermined toxicity. Here we used phage display to discover novel peptidic inhibitors of IDE, which were subsequently characterized *in vitro* and in cell culture assays. Among several peptide sequences tested, a cyclic dodecapeptide dubbed **P12-3A** was found to potently inhibit the degradation of insulin (K_i_ = 2.5 ± 0.31 μM) and other substrates by IDE, while also being resistant to degradation, stable in biological milieu, and highly selective for IDE. In cell culture, **P12-3A** was shown to potentiate several insulin-induced processes, including the transcription, translation and secretion of alpha-1 type I collagen in primary murine skin fibroblasts, and the migration of keratinocytes in a scratch wound migration assay. By virtue of its potency, stability, specificity for IDE, low cost of synthesis, and demonstrated ability to potentiate insulin-induced processes involved in wound healing and skin health, **P12-3A** holds significant therapeutic and cosmetic potential for topical applications.

## Introduction

Insulin is a pleiotropic peptide hormone that, although best known for its role in blood sugar regulation, is implicated in a wide array of physiological processes relevant to skin health and wound repair [1]. Insulin stimulates the proliferation [2, 3], differentiation [4] and migration [5, 6] of skin fibroblasts and keratinocytes, as well as the production and secretion of extracellular matrix (ECM) proteins, particularly collagen [7–13]. Conversely, all of these processes are impaired in the skin of mice with genetic deletion of the insulin receptor [14]. Moreover, impairments in wound healing and other skin disorders are common among patients with diabetes [15], a disease characterized by defects in insulin production or action.

Given the importance of insulin signaling to wound healing, topical insulin has been investigated in numerous studies in animals [6, 16–20] and humans [21], including several clinical trials [22–24]. However, the routine clinical use of topical insulin for wound management is not generally accepted as a first-line treatment, and significant adverse effects—including life-threatening hypoglycemia—have been reported [25].

Our group has been exploring an alternative approach to boosting insulin signaling that obviates the risk of hypoglycemia: namely, pharmacological inhibition of insulin-degrading enzyme (IDE) [26], the principal protease implicated in the catabolism and inactivation of insulin [27]. IDE inhibitors have been shown to potentiate insulin action in cultured cells [28] and in vivo [29–31]. Recently developed, highly selective IDE inhibitors exhibited potent antidiabetic properties [29], effects that were attributable to reduced catabolism of insulin. Importantly, mice with genetic deletion of IDE are viable [32–34]; thus—unlike insulin—IDE inhibitors possess no intrinsic risk of triggering life-threatening hypoglycemia. IDE is expressed to high levels in skin [35, 36] and—notably—is especially abundant in wound fluid [37, 38] where it degrades insulin [37, 38]. Thus, topical application of IDE inhibitors is strongly predicted to enhance insulin signaling in skin.

Although a number of IDE inhibitors have been developed [28, 29, 39–43], existing compounds are not ideal for topical applications due to their high cost of synthesis and undetermined toxicity. To overcome these limitations, we sought here to develop peptidic inhibitors of IDE that, by their intrinsic nature, would be inexpensive to manufacture and unlikely to be toxic. To that end, we used phage display to discover cyclic and linear peptide sequences that bind with high affinity to IDE. Among the sequences analyzed, a dodecameric, cyclic peptide dubbed **P12-3A**, proved to be a potent inhibitor of IDE that was stable in biologic milieu and highly selective for IDE. **P12-3A** was found to potentiate a number of insulin-stimulated processes in cultured skin cells, including collagen production in fibroblasts and migration of keratinocytes in a scratch wound assay. Given its high potency, selectivity for IDE, minimal potential for toxicity, and its low cost of manufacture, **P12-3A** possesses the characteristics needed to further explore the therapeutic and cosmetic potential of topical IDE inhibition.

## Results

**Fig 1:**
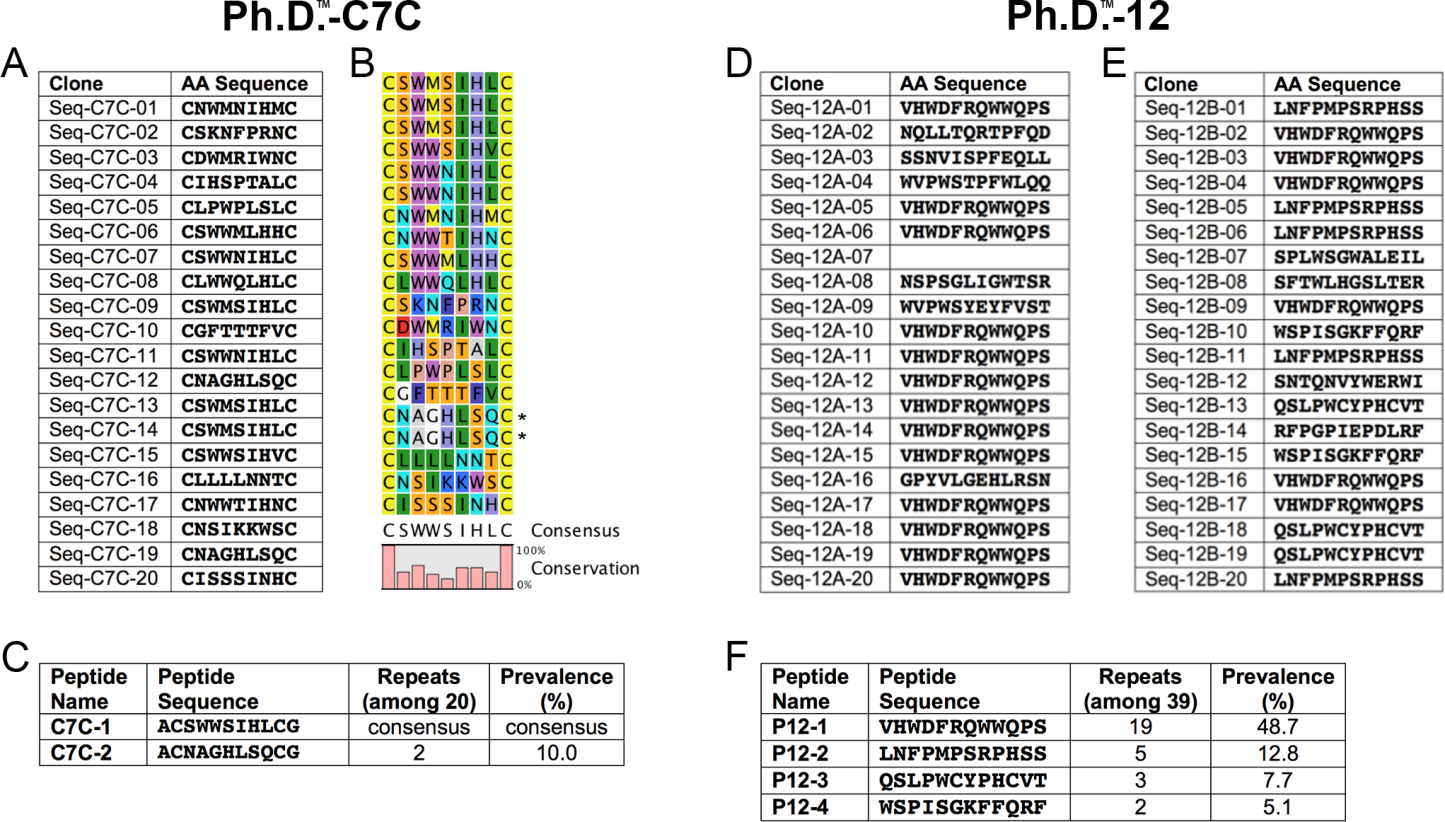
Peptides derived by phage display. ***A***, Peptide sequences deduced from DNA sequencing of 20 clones from the Ph.D.™-C7C library. ***B***, Consensus sequence derived from analysis of all data. ***C***, Parent peptides selected for synthesis and testing. ***C***,***D***, Peptide sequences deduced from DNA sequencing of 39 clones from the Ph.D.™-12 library, conducted as two independent runs (***D*** and ***E***). Note that Seq-12A-07 did not yield a decipherable sequence. ***F***, Parent peptides selected for subsequent synthesis and testing based on prevalence.

To identify novel peptidic inhibitors of IDE, we utilized phage display technology [44] to search for sequences that bind with high affinity to immobilized recombinant human IDE. Reasoning that IDE possesses an intrinsic affinity for cyclic peptides, we screened a library of cyclic peptides (Ph.D.^TM^-C7C, New England Biolabs) comprised of essentially all combinations of seven natural amino acids flanked by two cysteines (ACXXXXXXXCGGG…, where X represents any amino acid). The two cysteines form a disulfide bond that cyclizes each peptide, the alanine serves to protect the N-terminal cysteine from off-target interactions, and the three glycines form a flexible linker with the bacteriophage coat protein. Three rounds of panning were conducted using immobilized recombinant human IDE, with elution performed by addition of excess insulin in order to enrich for sequences that bind to the internal chamber of IDE. Twenty clones were selected for DNA sequencing, yielding sixteen unique amino acid sequences, some of which appeared more than once (Fig 1A). From analysis of all sequences (Fig 1B), a clear consensus sequence emerged (ACSWWSIHLCGGG…). This sequence, dubbed **C7C-1** (Fig 1C, Panel A in S1 Fig), was selected for subsequent synthesis and testing, together with another that appeared two times (ACNAGHLSQCGGG…), dubbed **C7C-2** (Fig 1C, Panel B in S1 Fig).

A second library of dodecapeptides (Ph.D.^TM^-12, New England Biolabs), consisting of ~10^9^ possible combinations of twelve amino acids, was also screened, in this case two separate times, yielding the sequences in Fig 1D and 1E. In this case, four sequences appeared multiple times (dubbed **12–1**, **12–2**, **12–3** and **12–4**), and these dodecapeptides were selected for subsequent synthesis and testing (Fig 1F, Panels C-F in S1 Fig).

The six selected peptide sequences (**C7C-1**, **C7C-2**, **12–1**, **12–2**, **12–3** and **12–4**) were synthesized, together with a variety of modifications, yielding a total of 25 peptides (Table 1). For the 12-mer peptides, we synthesized variants of the parent peptides truncated at one or both termini, to test whether the entire sequence was necessary or not, in the hope that shorter, and thus less expensive, sequences might be effective. Other modifications included N-terminal acetylation and C-terminal amidation, included to mimic the charge state of the peptides when incorporated in the phage coat protein (Table 1). To assess the extent to which these peptide sequences inhibit IDE, we quantified their potency in protease activity assays using recombinant human IDE and two different substrates: FRET1, a fluorogenic peptide [45], and recombinant human insulin. Peptides were initially tested at a few concentrations (10 μM, 100 μM and/or 500 μM) then, for those peptides showing good potency, dose-response relationships were obtained to quantify IC_50_ values, which were subsequently converted to inhibitory constants (K_i_ values) with the Cheng-Prusoff equation [46].

**Table 1:** Peptide sequences synthesized and their potency in activity assays with FRET1 and insulin.

Name	N-term	Sequence	C-term	K_i FRET1_ (μM)	K_i Insulin_ (μM)
**C7C-1**	NH_3_^+^	**ACSWWSIHLCG**	COO^-^	112 ± 6	3.7 ± 0.7
**C7C-1A**	NH_3_^+^	**ACSWWSIHLCG**	amide	>100	>10
**C7C-1B**	NH_3_^+^	**ACSWWSIHLCGGG**	COO^-^	>100	>10
**C7C-2**	NH_3_^+^	**ACNAGHLSQCG**	COO^-^	>500	>10
**C7C-2A**	NH_3_^+^	**ACNAGHLSQCG**	amide	>500	>10
**P12-1**	NH_3_^+^	**VHWDFRQWWQPS**	COO^-^	7.7 ± 0.7	0.8 ± 0.04
**P12-1A**	NH_3_^+^	**VHWDFRQWWQPS**	amide	5.0 ± 0.3	1.3 ± 0.2
**P12-1B**	NH_3_^+^	**VHWDFRQW**	amide	41 ± 6.4	7.0 ± 1.7
**P12-1C**	acetyl	**FRQWWQPS**	COO^-^	139 ± 21	>10
**P12-1D**	acetyl	**WDFRQWWQ**	amide	140 ± 28	>10
**P12-2**	NH_3_^+^	**LNFPMPSRPHSS**	COO^-^	>100	>10
**P12-2A**	NH_3_^+^	**LNFPMPSRPHSS**	amide	>100	>10
**P12-2B**	NH_3_^+^	**LNFPMPSR**	amide	>500	>10
**P12-2C**	acetyl	**MPSRPHSS**	COO^-^	>500	>10
**P12-2D**	acetyl	**FPMPSRPH**	amide	>500	>10
**P12-3**	NH_3_^+^	**QSLPWCYPHCVT**	COO^-^	8.9 ± 0.3	3.9 ± 1.6
**P12-3A**	NH_3_^+^	**QSLPWCYPHCVT**	amide	10 ± 0.4	4.1 ± 0.3
**P12-3B**	NH_3_^+^	**QSLPWCYP**	amide	39 ± 20	>10
**P12-3C**	acetyl	**WCYPHCVT**	COO^-^	35 ± 4.7	>10
**P12-3D**	acetyl	**LPWCTPHC**	amide	102 ± 14	>10
**P12-4**	NH_3_^+^	**WSPISGKFFQRF**	COO^-^	3.9 ± 0.5	1.5 ± 0.3
**P12-4A**	NH_3_^+^	**WSPISGKFFQRF**	amide	4.2 ± 1.3	2.6 ± 0.7
**P12-4B**	NH_3_^+^	**WSPISGKF**	amide	>500	>10
**P12-4C**	acetyl	**SGKFFQRF**	COO^-^	>500	>10
**P12-4D**	acetyl	**PISGKFFQ**	amide	>500	>10

Among the cyclic peptides tested, **C7C-1** exhibited a modest K_i_ value of 112 ± 6 μM against FRET1 but yielded considerably improved potency against insulin (K_i_ = 3.7 ± 0.7 μM) (Table 1). None of the other **C7C-1** derivatives exhibited K_i_ values below 100 μM for FRET1 or 10 μM for insulin, nor did **C7C-2** or its amidated derivative (Table 1).

Relative to the cyclic peptides **C7C-1** and **C7C-2** and their derivatives, the unmodified linear peptide **P12-1** exhibited significantly lower K_i_ values against FRET1 (7.7 ± 0.7 μM) as well as insulin (0.8 ± 0.04 μM) (Table 1). The C-terminally amidated version of **P12-1**, **P12-1A**, exhibited comparable K_i_ values against FRET1 and insulin (5.0 ± 0.3 μM and 1.3 ± 0.2 μM, respectively) (Table 1). Among three different 8-amino acid truncated versions of **P12-1**, the C-terminally truncated variant (**P12-1B**) exhibited slightly higher K_i_ values (41 ± 6.4 μM and 7.0 ± 1.7 μM for FRET1 and insulin, respectively), while the other N-terminally truncated (**P12-1C**) and N- and C-terminally truncated (**P12-1D**) variants exhibited relatively poor potency, with K_i_ values >100 μM for FRET1 and >10 μM for insulin (Table 1). These results suggest the N-terminal residues of **P12-1** (VHWD…) are the most critical determinants of its potency.

The dodecapeptide **P12-2** contained 3 proline residues (Table 1), which constrain the flexibility of the peptide backbone (Panel D in S1 Fig) and, in general, tend to render peptides less vulnerable to proteolytic degradation. However, **P12-2** and all its variants exhibited K_i_ values >100 μM for FRET1 and >10 μM for insulin (Table 1) and were not characterized further.

Peptide **P12-3** is noteworthy for containing two cysteine residues (Table 1), which are predicted to form a disulfide bond that cyclizes the peptide, together with 2 proline residues (Panel E in S1 Fig). The unmodified peptide, **P12-3**, and its C-terminally amidated variant, **P12-3A**, both potently inhibited the degradation of both FRET1 (K_i_ = 8.9 ± 0.3 μM and 10 ± 0.4 μM, respectively) and insulin (K_i_ = 3.9 ± 1.6 μM and 4.1 ± 0.3 μM, respectively) (Table 1). For the FRET1 substrate, the 8-amino acid truncated variants exhibited poorer potency, with the C-terminally (**P12-3B**) and N-terminally (**P12-3C**) truncated variants exhibiting of K_i_ values of 39 ± 20 μM and 35 ± 4.7 μM, respectively, and the dual N- and C-terminally truncated variant (**P12-3D**) exhibiting even higher K_i_ values of 102 ± 14 μM (Table 1). When insulin was used as a substrate, none of the truncated variants of **P12-3** exhibited K_i_ values <10 μM.

For the final peptide series, **P12-4** and its derivatives (Table 1, Panel F in S1 Fig), the full-length unmodified (**P12-4**) and amidated (**P12-4A**) versions showed good potency against FRET1 (K_i_ = 3.9 ± 0.5 μM and 4.2 ± 1.3 μM, respectively) and insulin (K_i_ = 1.5 ± 0.3 μM and 2.6 ± 0.7 μM, respectively), while none of the truncated variants (**P12-4B**, **P12-4C** and **P12-4D**) exhibited K_i_ values below 500 μM or 10 μM for FRET1 or insulin, respectively (Table 1).

**Fig 2:**
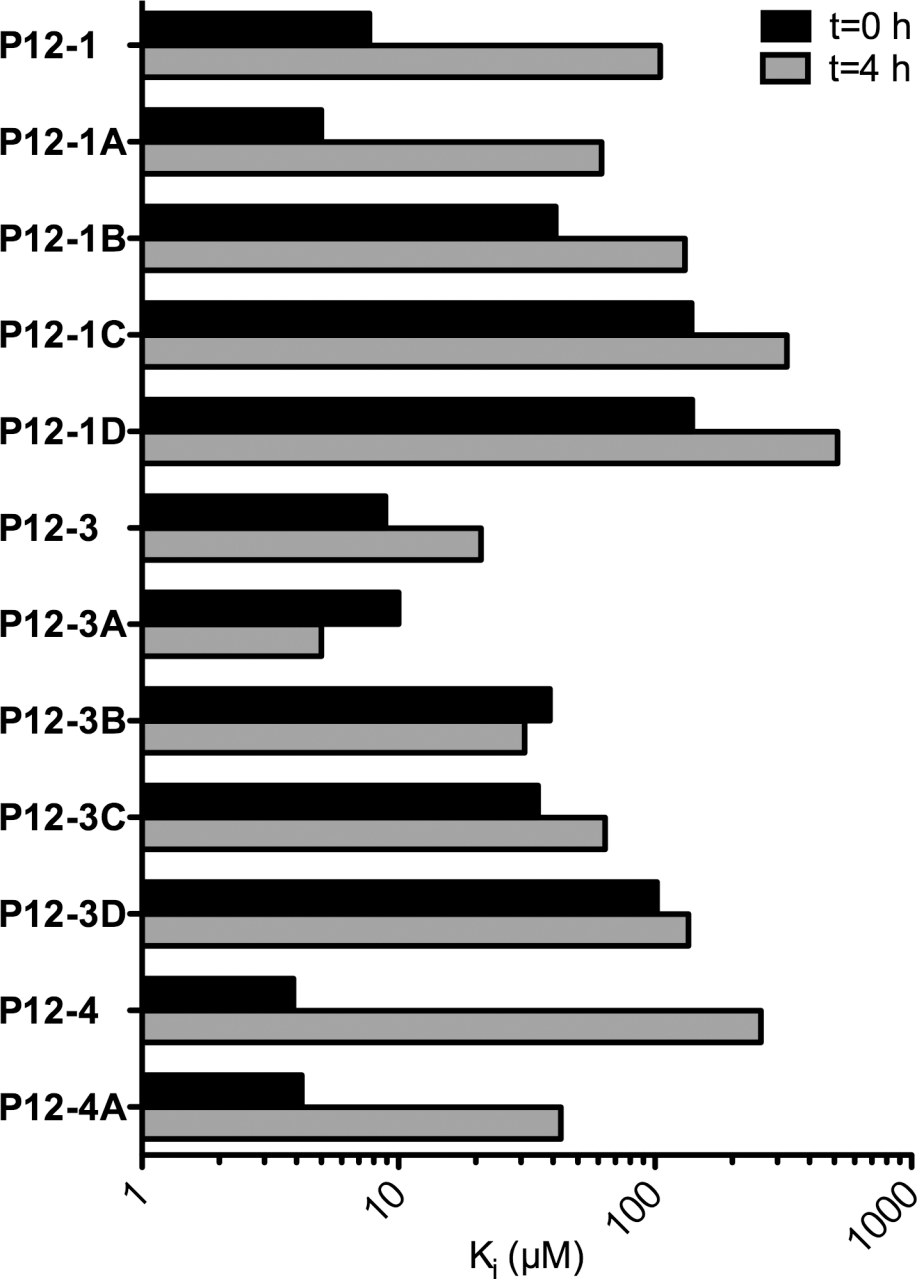
Vulnerability of peptides to degradation by IDE assessed by activity assays. IC_50_ values obtained for selected peptides pre-incubated with IDE 0 or 4 h before testing with the FRET1 assay. Note that all peptides except **P12-3A** and **P12-3B** showed reductions in apparent potency after prolonged incubation with IDE, reflecting degradation. Data are the average of duplicate assays that did not differ by more than 5%.

The sequences exhibiting high potency could be used either as conventional peptides—a preferred outcome due to their low cost of synthesis and low intrinsic toxicity—or, instead, as the starting point for the development of derivatives containing modifications that confer resistance to degradation (e.g., D-amino acids, beta-amino acids, etc.). To determine which peptides were susceptible to degradation by IDE, we incubated twelve peptides showing quantifiable inhibitory potency together with IDE for an extended period (4 h), then the potency of each was determined and compared to the potency immediately after addition of the enzyme (0 h). As shown in Fig 2, most peptides exhibited significant reductions in potency (i.e., increases in K_i_ values) after a 4-h incubation with IDE, reflecting proteolytic degradation by IDE. Notable exceptions to this trend included **P12-3A** and **P12-3B**, particularly **P12-3A** (QSLPWCYPHCVT-amide).

**Fig 3:**
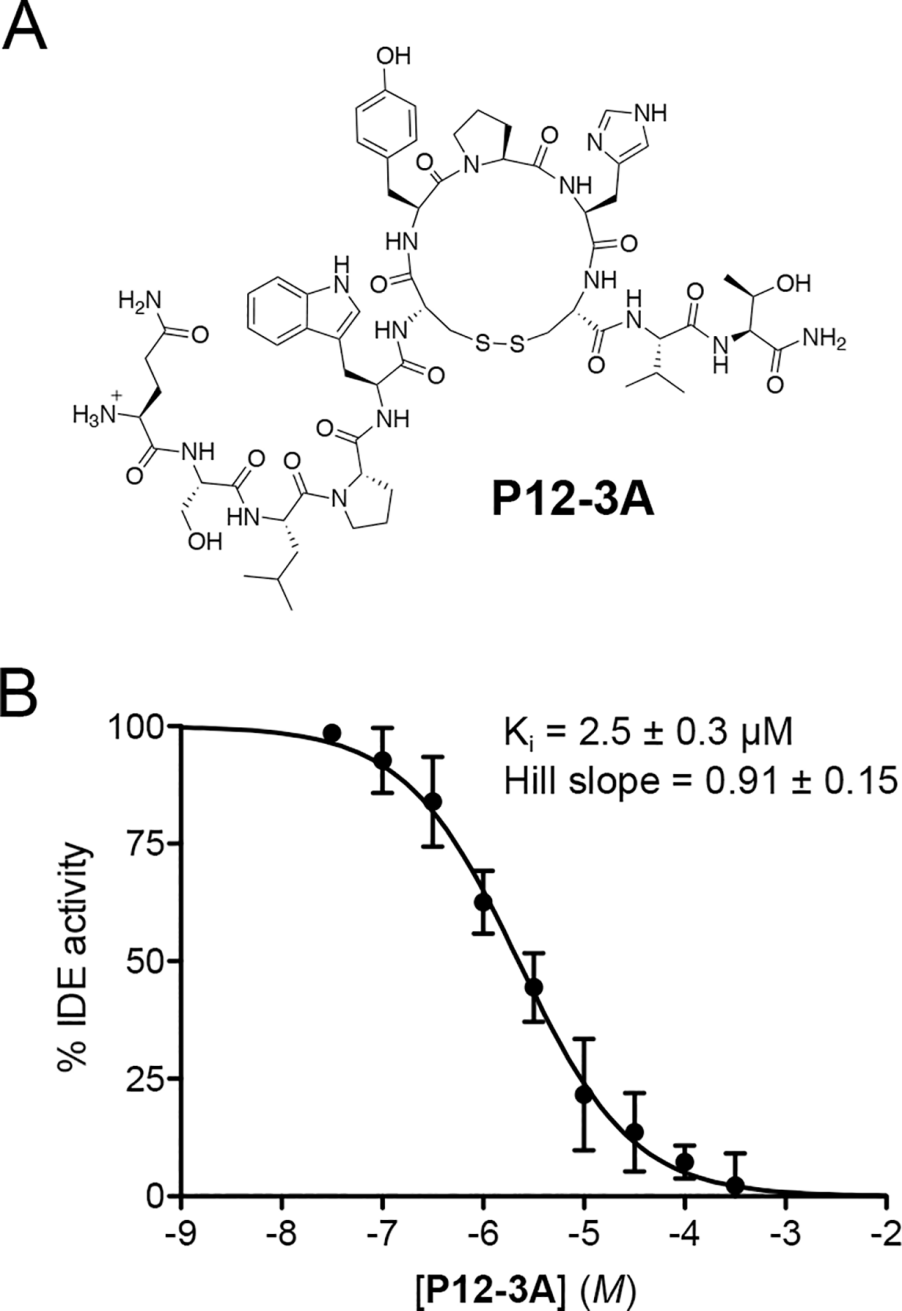
Structure and activity of P12-3A. ***A***, Structure of cyclized **P3-12A**. ***B***, Dose-response of **P12-3A** against insulin degradation by IDE. Data are mean ± SEM of 5 independent experiments.

Taken together with the potency of all peptides against insulin (Table 1), we concluded that **P12-3A** represented the best inhibitor for further studies, and a highly purified, deliberately cyclized version (Fig 3A) was synthesized. This cosmetic-grade version of **P12-3A** was found to be soluble up to ~500 μM in assay medium (PBS/0.05%BSA) and up to ~100 μM in cell culture medium, and was confirmed to exhibit similar potency against insulin degradation (K_i_ = 2.5 ± 0.31 μM, n = 5) (Fig 3B), and highly consistent inhibition constants were also observed for the degradation of two other substrates, FRET1 (K_i_ = 2.7 ± 0.50 μM, n = 6) and amyloid ß-protein (Aß) (K_i_ = 2.1 ± 0.34 μM, n = 6). Notably, **P12-3A** exhibited essentially no inhibition against 15 different proteases tested (S4 Fig), suggesting it is highly selective for IDE. Based on its potency, stability and selectivity for IDE, **P12-3A** was selected for use in downstream assays.

**Fig 4:**
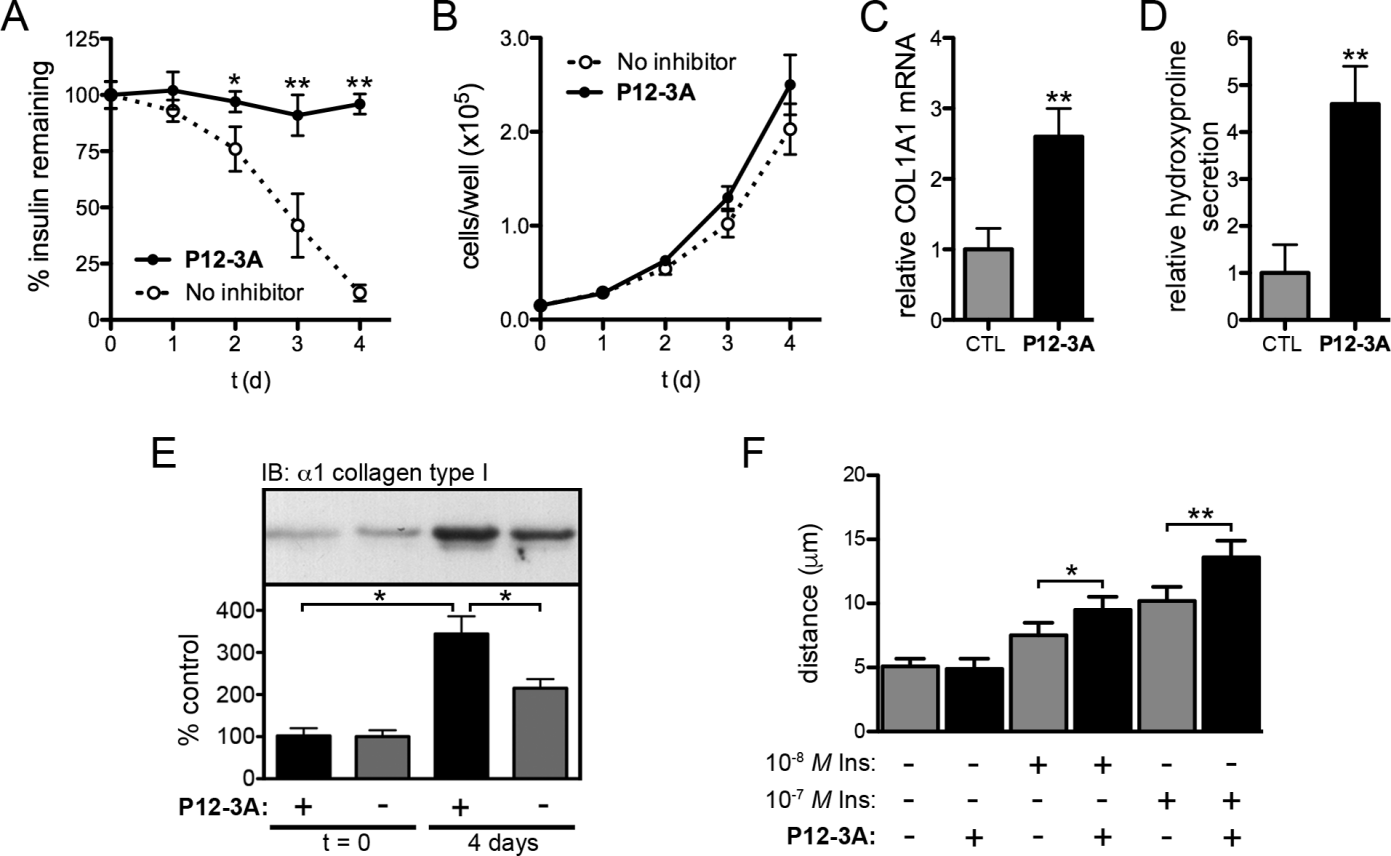
Effects of P12-3A on cultured skin cells. ***A***, Insulin concentrations as a function of time in logarithmically growing primary murine skin fibroblasts in the absence or presence of **P12-3A** (100 μM). Note that insulin levels remain constant in the presence of **P12-3A**, reflecting both the effectiveness of the peptide inhibiting insulin degradation and also the stability of the peptide in biological milieu. Data are mean ± SEM of 4 independent replications. *P<0.*05*, **P<0.*01*. ***B***, Proliferation of cells in the absence or presence of **P12-3A** (100 μM). Data are mean ± SEM of 6 independent replications. No significant differences were observed. ***C***,***D***,***E***, **P12-3A** (100 μM) potentiates insulin-induced collagen production in skin fibroblasts. Collagen production was assessed by *COL1A1* mRNA levels (***C***), levels of hydroxyproline secreted into the medium (***D***), and cell-associated mature alpha-1 type I collagen levels detected by Western blotting (***E***). Data are mean ± SD of 4 independent replications. *P<0.*05*, **P<0.*01*. ***F***, **P12-3A** (100 μM) potentiates the migration of keratinocytes in a scratch wound assay. Migration of HaCaT cells 48 h after induction of a scratch wound in the presence of the indicated quantities of insulin and/or **P12-3A** (100 μM). Data are mean ± SEM of 6 independent replications. *P<0.*05*, **P<0.*01*.

Insulin promotes wound healing and overall skin health by affecting a number of processes, including cell proliferation [2, 3], cell migration [5, 6], and the production and secretion of ECM components, particularly type I collagen [7–13]. We therefore assessed the ability of **P12-3A** to influence these processes in cultured skin fibroblasts and keratinocytes. To that end, primary mouse skin fibroblasts were grown for 4 d in multiple concentrations of insulin in the presence or absence of **P12-3A,** and insulin concentration over time was monitored by ELISA. In untreated cells, 10 nM insulin was degraded approximately 90% by day 4 in logarithmically growing cells, whereas insulin levels remained constant in cells treated with either 100 μM **P12-3A** (Fig 4A). These results demonstrate that **P12-3A** is effective at inhibiting insulin degradation by skin fibroblasts and, moreover, confirm that the compound is stable in biological milieu. Relative to control cells, cell proliferation in the presence of 10 nM insulin was found to be modestly increased in the presence of 100 μM **P12-3A** (Fig 4B), but this did not achieve statistical significance. Finally, the effects of **P12-3A** on collagen production in were assessed at both the transcriptional and the posttranslational level in confluent monolayers of fibroblasts. After 4 d of treatment with **P12-3A** (100 μM), mRNA levels for the major form of collagen, alpha-1 type I collagen (*COL1A1*), were quantified by RT-PCR and found to be increased to ~2.6 times the levels of untreated cells (Fig 4C). To assess overall levels of collagen production and secretion, we quantified levels of hydroxyproline, a modified amino acid present almost exclusively in collagen proteins [47]. In the presence of **P12-3A**, hydroxyproline levels secreted into the medium were found to be increased to levels ~4.6 times that secreted by untreated cells (Fig 4D). Western blotting also confirmed that mature, cell-associated collagen levels were increased in the presence of **P12-3A** (Fig 4E). Finally, using an *in vitro* scratch wound assay, **P12-3A** (100 μM) was found to result in statistically significant increases in the migration of keratinocytes in the presence of different concentrations of insulin (Fig 4F).

## Discussion

Although a variety of potent and selective IDE inhibitors have been developed [28, 29, 39–43], current inhibitors are difficult to synthesize, expensive to generate and/or contain chemical moieties or constituents with established or undetermined potential for toxicity. Due to these and other considerations, existing IDE inhibitors are poorly suited for topical applications. To overcome these limitations, in the present study we aimed to develop peptidic inhibitors of IDE suitable for use in wound healing or cosmetic applications. Peptides are easy to manufacture and therefore inexpensive to scale up and, being composed solely of all-natural amino acids, are unlikely to possess any degree of toxicity. To that end, we used phage display technology to select for a range of peptides that bind to IDE with strong affinity, which were then screened for resistance to degradation by IDE. One cyclic dodecapeptide in particular, **P12-3A**, proved to be a potent and stable inhibitor of IDE that showed excellent selectivity and also showed no evidence of toxicity in cell culture experiments. Critically, **P12-3A** was found to potentiate a number of insulin-stimulated processes relevant to wound healing and skin health, including collagen production by fibroblasts and migration of keratinocytes in response to scratch wounds.

Phage display proved to be a highly effective approach for developing peptidic inhibitors. From among six parent peptides selected for further testing, four exhibited low-micromolar K_i_ values against insulin, and one (**P12-1**) exhibited sub-micromolar potency (K_i_ = 0.8 ± 0.04 μM). Among the modified versions of these parent peptides, an additional five exhibited K_i_ values <10 μM; thus nine of the twenty-five peptides tested (36%) showed good activity. These hit rates are markedly higher than those obtained through high-throughput compound screening [41, 45, 48] or other approaches [29]. Notably, the potency of **P12-1** (K_i_ = 800 nM) compares favorably to that of the highly optimized and extensively characterized macrocyclic IDE inhibitor, **6bK**, which shows a IC_50_ value of ~100 nM against insulin [29]. Given that **6bK** underwent considerable optimization [29], the potency of **P12-1**, a simple, unmodified dodecapeptide, is notable.

Although the majority of peptides showed good affinity for IDE, most were also degraded by it. Due to the peculiarities of its structure [26, 49–51], IDE is a pure peptidase that cannot degrade proteins; thus it is unsurprising that phage display would reveal sequences that bind strongly while attached to the bacteriophage coat protein but are nevertheless degraded when synthesized as a short peptide. The particular stability of **P12-3A** (and related peptides) likely derives from the fact that it contains two cysteines and can therefore form a cyclic peptide. Of note, it is unusual for cyclic peptides to emerge from a library of linear peptides, because cyclization tends to slow the maturation of the bacteriophage, leaving the phage expressing them at a competitive disadvantage when grown in parallel with phage expressing linear peptides. This suggests this peptide sequence was strongly favored during the selection process.

Based on the ability of **P12-3A** to potentiate insulin-stimulated collagen production and cell migration, topical IDE inhibition would appear to hold significant therapeutic potential in wound healing, particularly for diabetic patients [1]. Given the accruing evidence that insulin signaling pathways are critical for wound healing [1, 22] and, given that IDE is abundant in wound fluid [37, 38], where it actively degrades insulin, there is a strong prediction that pharmacological inhibition of IDE will promote wound healing [28]. This prediction is strongly supported by the finding that IDE inhibitors potentiate insulin action *in vivo* in part by preserving endogenous insulin [32, 33] and possibly *via* actions downstream of insulin receptor binding [28]. Importantly, by contrast to direct topical administration insulin, which can cause life-threatening hypoglycemia [25], pharmacological inhibition of IDE possesses no intrinsic risk of triggering hypoglycemia [32–34].

One of the most immediately implementable potential cosmetic applications for **P12-3A** may be as an adjuvant for microneedling procedures [52]. Also known as percutaneous collagen induction [53, 54], microneedling is a minimally invasive, widely used technique by which production of ECM proteins in the dermis can be stimulated by introducing uniform, sterile wounds in a controlled manner [52]. Although originally developed for skin rejuvenation, this technique is now being used as a novel treatment for a wide range of cosmetic and medical conditions, including acne, alopecia, stretch marks, hyperhidrosis and scarring of multiple types [52, 53]. Topical application of **P12-3A** prior to microneedling would permit the delivery of the peptide subcutaneously [52], thus maximizing its impact on the processes involved in wound repair.

In sum, using phage display technology, we have generated novel peptidic inhibitors of IDE that, by virtue of their low cost of synthesis and minimal risk of toxicity, have the properties needed to explore the therapeutic and cosmetic potential of topical IDE inhibition. Given the importance of insulin in wound healing and normal skin health, these novel inhibitors, as well as future derivatives thereof, will be useful for exploring the involvement of IDE in these processes, and may also hold significant value as adjuvants for medicinal and cosmetic treatments.

## Materials and methods

### Materials

Anti-alpha-1 type I collagen antibody (Cat. No. AB765P) and horseradish peroxidase (HRP)-conjugated anti-rabbit IgG antibody (Cat. No. A0545) were from Sigma-Aldrich (St. Louis, MO, USA). Anti-glyceraldehyde-3-phosphate dehydrogenase (GAPDH; Cat. No. AF5718) antibody was from R&D Systems (Minneapolis, MN, USA). HRP-conjugated anti-goat IgG antibody (Cat. No. sc-2354) was from Santa Cruz Biotechnology (Santa Cruz, CA, USA). Materials for Western blotting and cell culture were from Thermo Fisher Scientific (Waltham, MA, USA). Insulin ELISAs (Cat. No. 90082) were from Crystal Chem (Downers Grove, IL, USA). Primary murine skin fibroblasts were a generous gift from Dr. Jorge Busciglio (UC Irvine). HaCaT cells and optimized growth medium were purchased from AddexBio Technologies (San Diego, CA, USA). Unless specified, all other reagents were from Sigma-Aldrich (St. Louis, MO, USA).

### Phage display

The selection of IDE-binding peptide sequences was conducted by phage display using the Ph.D.**™**-C7C and Ph.D.**™**-12 Phage Display Library Kits from New England Biolabs (Ipswich, MA, USA) according to manufacturer’s recommendations. Briefly, purified, glycerol-free, recombinant human IDE (100 μg/mL) [35] was immobilized onto Corning^®^ High Bind, 96-well, round-bottom plates (Cat. No. CLS3366). After washing and prior to addition of bacteriophage, activity assays with FRET1 (see below) were used to confirm the presence of proteolytically active IDE in wells coated in parallel with those used for panning. Three rounds of panning were conducted, with 2 x 10^11^ phage/well added at each step. After incubation at room temperature for 60 min, bound phage were eluted by addition of excess recombinant human insulin (100 μg/mL) and amplified for the subsequent round of panning. After the third round of panning, the eluate was titered and individual clones were selected for DNA sequencing. Peptide sequences were decoded and consensus sequences searched for using CLC Sequence Viewer (Version 7.5).

### Peptide synthesis

Peptides were synthesized by automated solid-phase peptide synthesis by Sigma-Aldrich, with the exception of **C7C-1**, which was synthesized in-house essentially as described [28] and analyzed by electrospray-ionization mass spectrometry (ESI-MS; S2 Fig) and HPLC (S3 Fig). Cosmetic-grade, gram-scale quantities of **P12-3A** were synthesized by GenScript Biotechnology Corp. (Piscataway Township, NJ, USA).

### Degradation assays

IDE activity was quantified by monitoring the degradation of Mca-GGFLRKVGQK(Dnp) (FRET1, 5 μM) [49, 55], fluoresceinated and biotinylated amyloid ß-protein (FAßB; 500 nM) or recombinant human insulin (50 nM) in PBS supplemented with 0.05% BSA. FRET1 degradation was measured by changes in fluorescence (**λ**_ex_ = 340 nm, **λ**_em_ = 420 nm); FAßB degradation was monitored by fluorescence polarization (**λ**_ex_ = 488 nm, **λ**_em_ = 525 nm), as described [56]; and insulin degradation was quantified by ELISA. *In vitro* activity assays incorporated recombinant human IDE (1 nM) purified from bacteria [35]. For quantitation of insulin degradation in primary murine skin fibroblasts, cells (1 x 10^5^/well) were plated in 96-well plates and maintained in DMEM supplemented with 10% fetal bovine serum (FBS), 2mM glutamine, penicillin and streptomycin. After addition of insulin (10 nM or 100 nM), samples of conditioned medium were removed daily and quickly frozen, then insulin levels were quantified in parallel by ELISA according to manufacturer’s recommendations (Crystal Chem, Downers Grove, IL, USA). Assessment of the activity of **P12-3A** against a variety of matrix-metalloproteases was conducted using the Matrix Metalloproteinase (MMP) Inhibitor Profiling Kit, Fluorometric RED (Enzo Life Sciences, Inc., Farmingdale, NY, USA) according to manufacturer’s recommendations using the broad-spectrum MMP inhibitor, NNGH, as a positive control. Activity assays on additional proteases were conducted using the FAßB degradation assay, using a custom protease inhibitor cocktail (PIC) comprised of cOmplete™, Mini, EDTA-free Protease Inhibitor Cocktail supplemented with 1,10-phenanthroline (2 mM) and pepstatin A (5 μM).

### Cell proliferation

Primary murine skin fibroblasts cells (1 x 10^5^/well) were plated in 96-well plates, using separate plates for each timepoint and endpoint. Cell proliferation was quantified using the CellTiter 96® AQueous Non-Radioactive Cell Proliferation Assay (Promega Corp., Madison, WI, USA) according to manufacturer’s recommendations.

### RNA quantification

RNA was extracted from freshly lysed cells, reverse transcribed and amplified using the Ambion^®^ Fast SYBR^®^ Green Cells-to-C_T_™ Kit according to manufacturer’s recommendations (Thermo Fisher Scientific, Waltham, MA, USA). The quantitative real-time PCR reaction was conducted using a 7500 real-time PCR system and analyzed using System SDS software v2.0.5 (Applied Biosystems). Murine COL1A1 mRNA was detected using the following primers (forward: 5’-ACCTAAGGGTACCGCTGGA and reverse: 5’ TCCAGCTTCTCCATCTTTGC). Fold change differences between samples were determined using the comparative C_t_ (ΔΔC_t_) method, normalized to internal standards detected with the SYBR^®^ Green Cells-to-C_T_™ Control Kit according to manufacturer’s recommendations (Thermo Fisher Scientific, Waltham, MA, USA) calculated by 2^-ΔΔCt^.

### Hydroxyproline quantification

For quantitation of hydroxyproline secretion by primary murine skin fibroblasts, cells (1 x 10^5^/well) were plated in 24-well plates DMEM supplemented with 10% FBS, 2mM glutamine, penicillin, streptomycin and 100 nM insulin, in the absence or presence of 100 μM **P12-3A**. After incubation for 4 days, the conditioned medium was removed, centrifuged at 1000 x g for 10 min to remove cellular debris, and hydroxyproline levels were quantified using the Hydroxyproline Assay Kit according to manufacturer’s recommendations (Sigma-Aldrich, St. Louis, MO, USA).

### Western blotting

Protein was collected using the M-Per Mammalian Extraction Reagent and the concentration was quantified using the Pierce™BCA Protein Assay Kit according to manufacturer’s recommendations (Thermo Fisher Scientific, Waltham, MA, USA). Protein (30 μg/well) was separated SDS-PAGE under reducing conditions using Novex™ 10% polyacrylamide tris-glycine mini gels and transferred to nitrocellulose membranes as described [57]. Briefly, membranes were blocked in 5% non-fat milk in tris-buffered saline supplemented with 0.2% Tween-20 (TBST), cut into segments and incubated for 1 h at room temperature with anti-alpha-1 type I collagen (1:5000) and anti-GAPDH (1:10,000) antibodies, washed extensively in TBST, then probed with anti-rabbit (1:20,000) or anti-goat (1:50,000) secondary antibodies, respectively, and detected by enhanced chemoluminescence using SuperSignal West Pico Substrate. Protein expression, normalized to GAPDH levels, was quantified using the band analysis tools of ImageLab software, version 4.1 (Bio-Rad Laboratories, Inc., Hercules, CA, USA).

### *In vitro* scratch wound assay

Cell migration in HaCaT cells after induction of scratch wounds was quantified essentially as described [58]. Briefly, HaCaT cells maintained in optimized DMEM (AddexBio Technologies, San Diego, CA, USA) supplemented with 10% FBS, penicillin and streptomycin, were grown to confluency in 24-well tissue culture plates, and scratch wounds were induced with a 200-μL pipette tip. After growth for 48 h in the absence or presence of insulin (10 nM or 100 nM) and/or **P12-3A** (100 μM), cell migration distance was quantified by two independent, blinded observers using a Nikon TMS inverted light microscope (Nikon Corp., Melville, NY, USA) fitted with a ruler reticle.

### Statistical analyses

Tests for statistical significance were performed by using the two-tailed Student's *t* test with various levels of significance (*P* = 0.05, 0.01). For comparisons with unequal numbers of replications per group, Hartley's *F*_max_ was calculated to check for homogeneity of variance. All calculations and curve fitting were performed in Prism for Mac OS X, version 5.0b (GraphPad Software, Inc., La Jolla, CA, USA).

## Supporting information

S1 Fig Structures of parent peptides discovered by phage display. Note that **P12-3**, although derived from a library of primarily linear peptides, is predicted to be a cyclic peptide.
(TIF)

S2 Fig Confirmation of mass of C7C-1 by ESI-MS. The entire spectrum as well as expanded regions are shown.
(TIF)

S3 Fig Analysis of purity of C7C-1 by HPLC. Note that the purity is ~95%.
(TIF)

S4 Fig Selectivity of P12-3A for IDE vis-à-vis other proteases. Activity of **P12-3A** (100 μM) against (***A***) multiple matrix-metalloproteases (MMPs) and (***B***) multiple peptidases of different protease classes. Note that significant inhibition was observed exclusively for IDE, with modest inhibition (~18%) observed for just one of 15 other proteases tested (MMP-7). Data are mean ± SD, n = 8–16 per group. *P*<0.05 by 2-tailed Student’s t-test. Note that all positive controls (NNGH or protease inhibitor cocktail (PIC)) exhibited significant inhibition **(***P*< 0.01). See *Materials and Methods* for details. NEP, neprilysin; mCatD, murine cathepsin D; hCatD, human cathepsin D.
(TIF)

S1 Dataset DNA sequences from phage display screens. (ZIP)

S2 Dataset Source data for all quantitative results. (XLSX)
