# Supplementary figures and images for: Peptidic inhibitors of insulin-degrading enzyme with potential for dermatological applications discovered via phage display
Source: PLoS One. 2018 Feb 15;13(2):e0193101. doi: 10.1371/journal.pone.0193101 (PMC5814047; doi:10.1371/journal.pone.0193101)

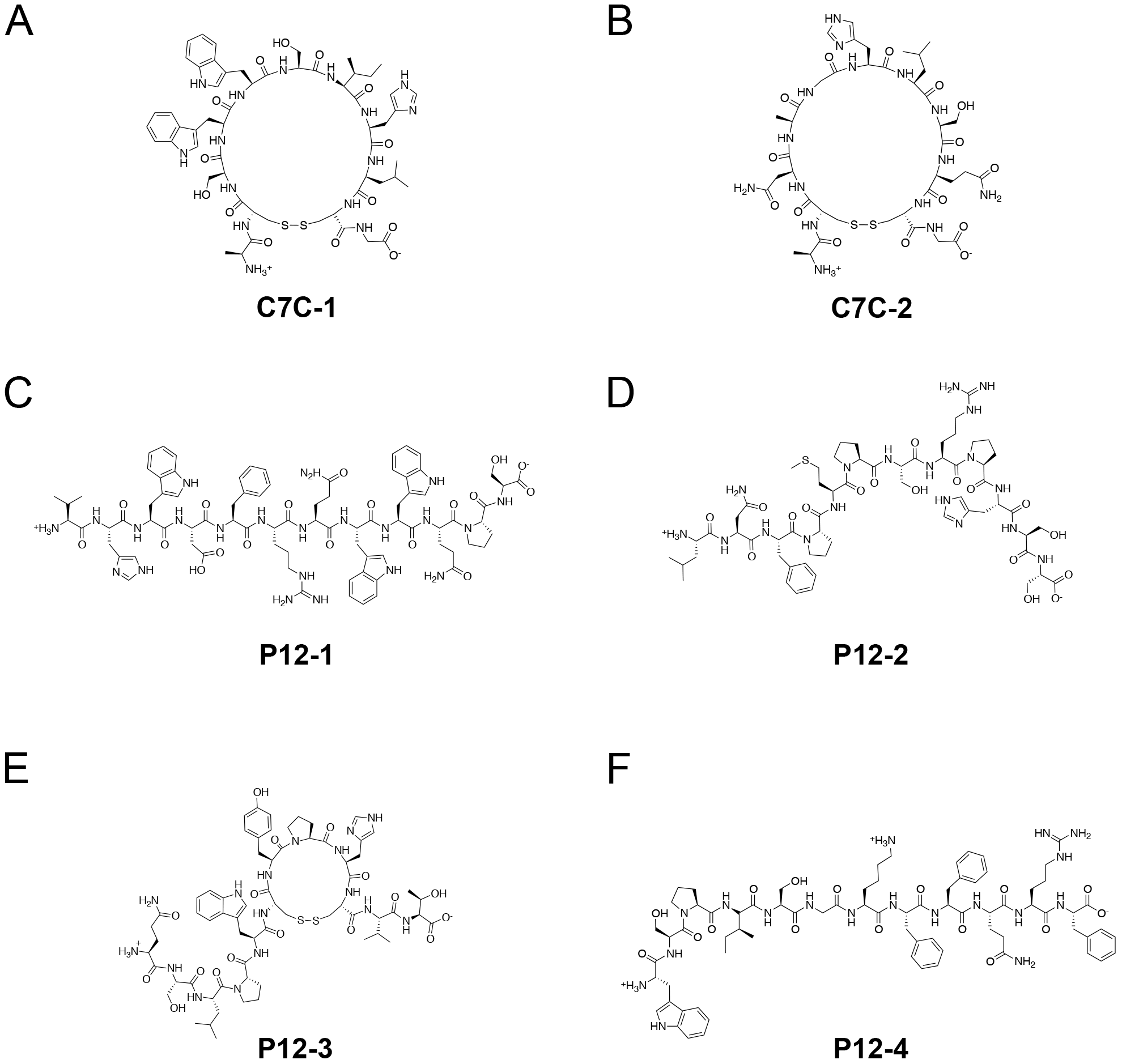

Supplement: S1 Fig — Note that P12-3, although derived from a library of primarily linear peptides, is predicted to be a cyclic peptide. (TIF) [file pone.0193101.s001.tif]

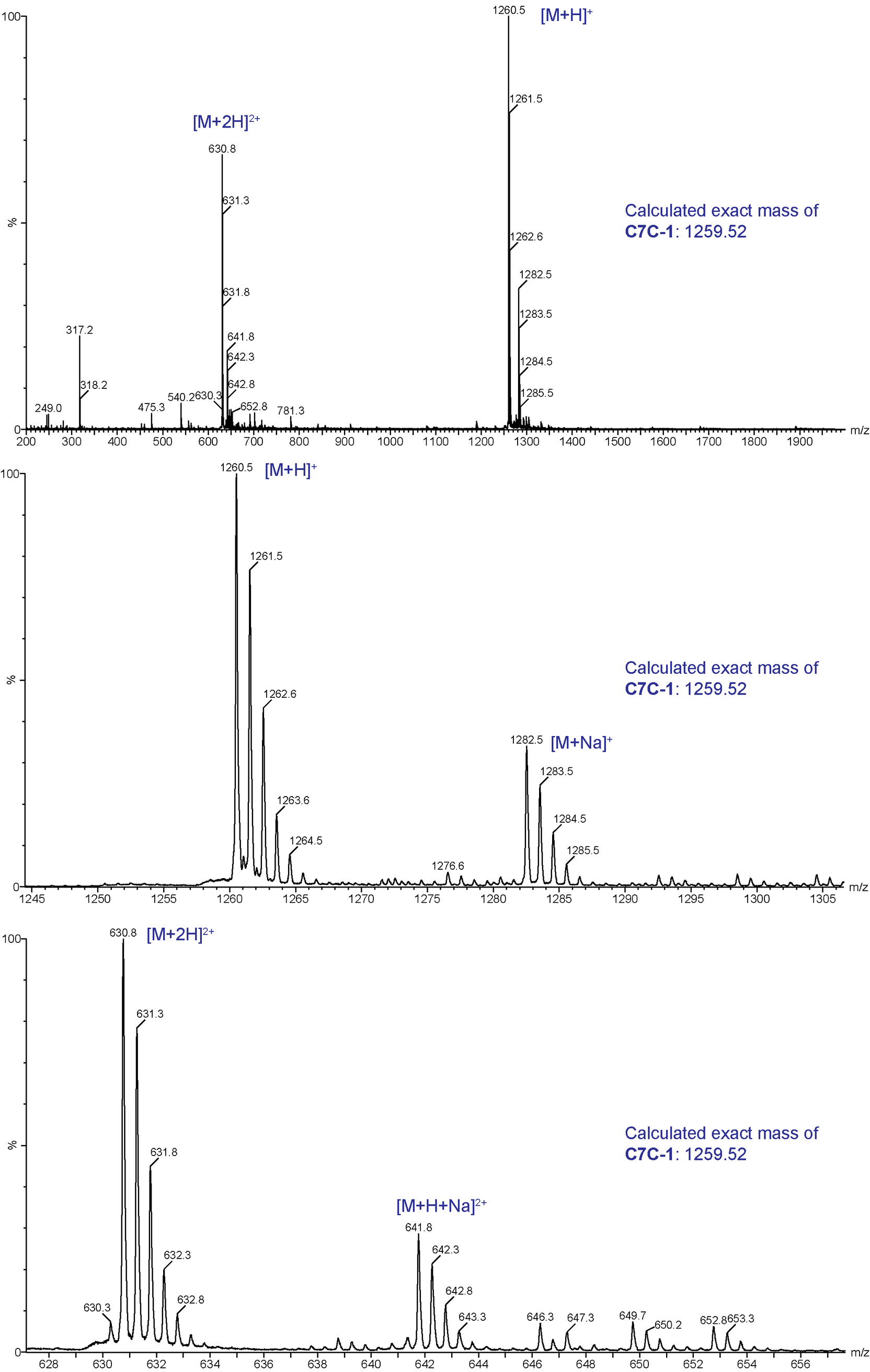

Supplement: S2 Fig — The entire spectrum as well as expanded regions are shown. (TIF) [file pone.0193101.s002.tif]

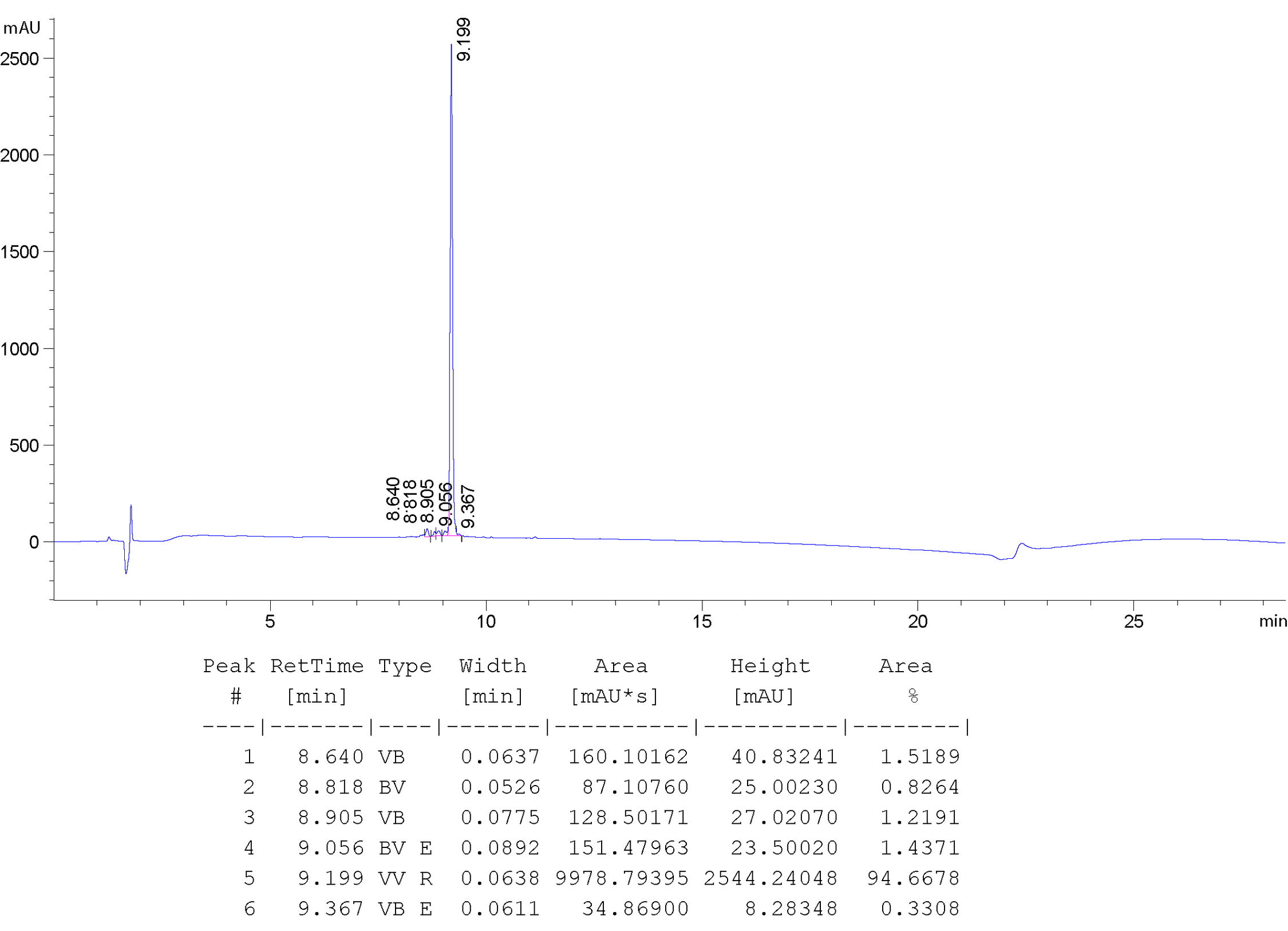

Supplement: S3 Fig — Note that the purity is ~95%. (TIF) [file pone.0193101.s003.tif]

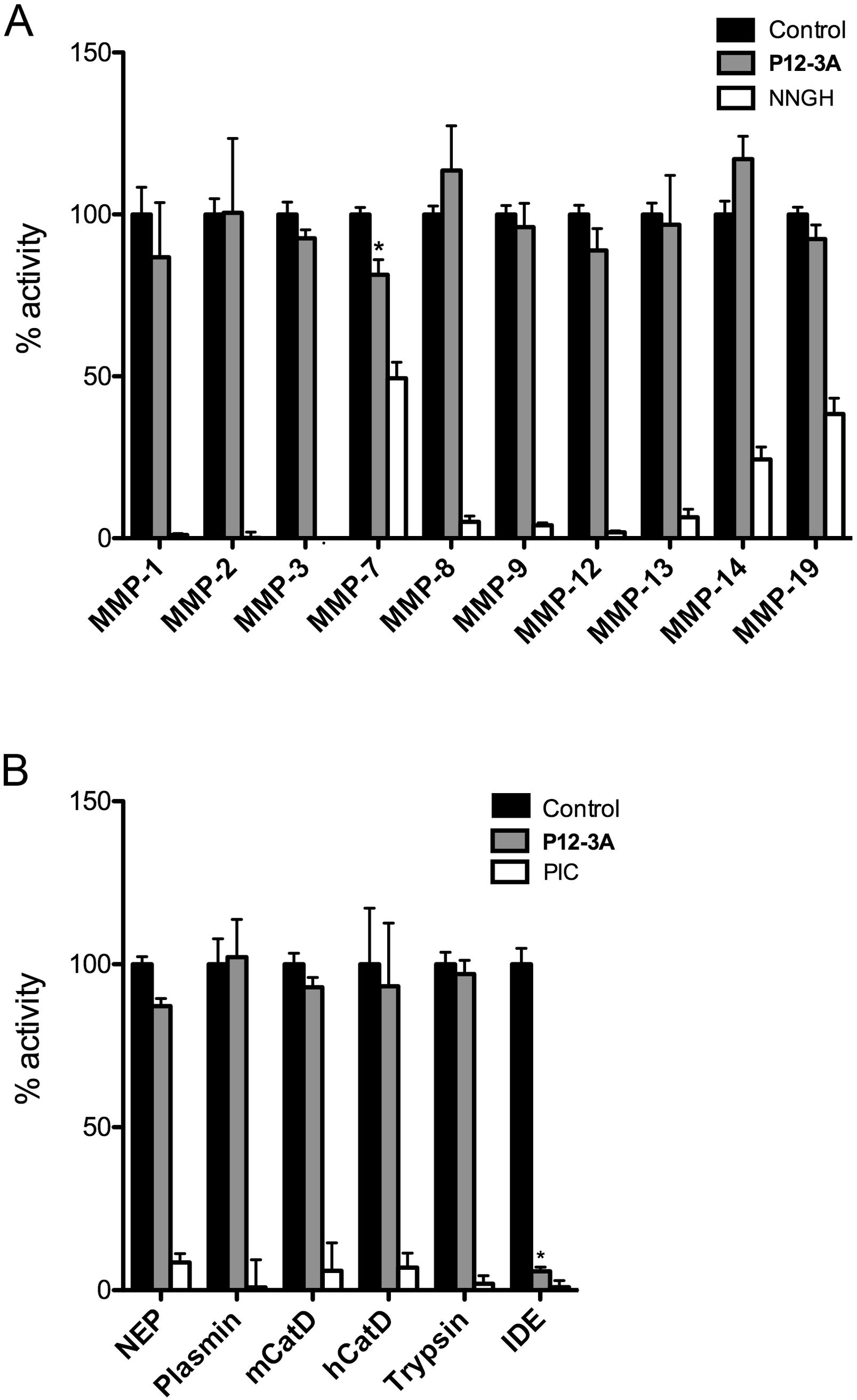

Supplement: S4 Fig — Activity of P12-3A (100 μM) against (A) multiple matrix-metalloproteases (MMPs) and (B) multiple peptidases of different protease classes. Note that significant inhibition was observed exclusively for IDE, with modest inhibition (~18%) observed for just one of 15 other proteases tested (MMP-7). Data are mean ± SD, n = 8–16 per group. P<0.05 by 2-tailed Student’s t-test. Note that all positive controls (NNGH or protease inhibitor cocktail (PIC)) exhibited significant inhibition (P< 0.01). See Materials and Methods for details. NEP, neprilysin; mCatD, murine cathepsin D; hCatD, human cathepsin D. (TIF) [file pone.0193101.s004.tif]
